# Knockdown of circular RNA VANGL1 inhibits TGF‐β‐induced epithelial‐mesenchymal transition in melanoma cells by sponging miR‐150‐5p

**DOI:** 10.1111/jcmm.16887

**Published:** 2021-11-09

**Authors:** Hongfeng Zhou, Jin Wu, Shaolong Leng, Chongchao Hou, Laiming Mo, Xue Xie, Ling Wang, Yunsheng Xu

**Affiliations:** ^1^ Department of Medical Oncology Harbin Medical University Cancer Hospital Harbin, Heilongjiang China; ^2^ Department of Dermatovenereology The Affiliated Hospital of Shenzhen University Shenzhen China

**Keywords:** circular RNA VANGL1, EMT, melanoma, miR‐150‐5p, TGF‐β

## Abstract

Melanoma is one of the most aggressive and life‐threatening skin cancers, and in this research, we aimed to explore the functional role of circular RNA VANGL1 (circVANGL1) in melanoma progression. The expression levels of circVANGL1 were observed to be significantly increased in clinical melanoma tissues and cell lines. Moreover, circVANGL1 knockdown suppressed, while circVANGL1 overexpression promoted the proliferation, migration and invasion abilities of melanoma cells. Further investigations confirmed the direct binding relation between circVANGL1 and miR‐150‐5p in melanoma, and restoration of miR‐150‐5p blocked the effects of circVANGL1 overexpression in melanoma cells. We further found that circVANGL1 was up‐regulated by TGF‐β treatment, and the enhanced EMT of TGF‐β‐treated melanoma cells was blocked by circVANGL1 knockdown. In conclusion, these results indicated that circVANGL1 might serve as a promising therapeutic target for melanoma.

## 1 INTRODUCTION

Melanoma is one of the most lethal cutaneous cancers with a highly aggressive and metastatic phenotype.^1^ The incidence of melanoma has risen dramatically over past decades. For early‐stage melanoma, surgical resection is still recognized as the mainstay of therapeutic method,^2^ but this disease has the proclivity to metastasize. At present, the long‐term survival rate for patients with metastatic melanoma remains unfavourable.^3^ Therefore, it is of great clinical significance to clarify the molecular mechanisms and genetic alterations underlying melanoma growth and metastasis.

More than 90% of transcripts from human genome are not translated into proteins.^4^ Circular RNAs (circRNAs), a large group of widespread and diverse endogenous non‐coding RNAs, are characterized by a covalently closed loop structure without a 5’ cap or a 3’ Poly A tail.^5^ circRNAs were first considered as products of splicing errors,^6^ but till now, emerging evidence indicates that they play important roles in many human diseases, including cancers. Considering their conserved sequences, stable structures and tissue‐specific expression, circRNAs were deemed to be promising biomarkers for cancer diagnosis.^7^ circRNA VANGL1 (circVANGL1) is generated from two exons of the Van Gogh‐like 1 (VANGL1) gene and serves as a tumour promoter in bladder cancer.^8^ Up‐regulated circVANGL1 also contributes to the progression of non‐small cell lung cancer.^9^ In this research, we aimed to explore the functional role of circVANGL1 in melanoma progression.

## 2 MATERIALS AND METHODS

### 2.1 Patients and tissue samples

Sixty‐nine melanoma tissues and twenty benign nevi tissues were obtained from patients who underwent surgical resection at the Seventh Affiliated Hospital of Sun Yat‐sen University. None of these patients underwent chemotherapy or radiotherapy prior to surgery. After collection, all tissue samples were immediately snap‐frozen in liquid nitrogen and stored at −80℃. This study was approved by the Ethics Committee of The Seventh Affiliated Hospital of Sun Yat‐sen University. Written informed consent was obtained from all subjects in accordance with the Declaration of Helsinki.

### 2.2 Cell culture and treatments

Human melanoma cell lines, including WM‐35, WM‐115 and A375, and normal human epidermal melanocyte HEMa‐LP cells were purchased from the Cell Bank of Chinese Academy of Sciences and maintained in RPMI‐1640 Medium (Thermo Fisher Scientific, Inc.) containing 10% foetal bovine serum (FBS; Invitrogen) and 1% penicillin/streptomycin at 37℃ in a humidified incubator with 5% CO_2_. The medium was changed every 48–72 h. For TGF‐β stimulation, cells were treated with 10 ng/ml recombinant TGF‐β (R&D Systems, Inc.) for 7 days.

For overexpression of circVANGL1 in cells, circVANGL1 cDNA was amplified and subcloned into the pcD‐ciR vector (Geneseed Biotech Inc.), which contains a front circular frame and a back circular frame. The empty vector was used as negative control. The small interfering RNA (siRNA) targeting circVANGL1 (si‐circVANGL1), the negative control siRNA (si‐NC), miR‐150‐5p mimics (miR‐150‐5p), negative mimics control (miR‐NC), miR‐150‐5p inhibitor (anti‐miR‐150‐5p) and negative inhibitor control (anti‐miR‐NC) were obtained from Guangzhou RiboBio Co., Ltd. Transfection was carried out using Lipofectamine 2000 (Invitrogen).

### 2.3 RT‐qPCR analysis

Total RNA was isolated using TRIzol reagent (Invitrogen) and then treated with RNase‐free DNase I (Promega). The RNA was first reversely transcribed to cDNA using the PrimeScript™ RT reagent kit (TaKaRa). Thereafter, qPCR reactions were carried out using a SYBR Green PCR Kit (TaKaRa) on a 7500HT Real‐Time PCR System (Applied Biosystems). The 2^−ΔΔCt^ method was used to calculate the relative gene expression,^10^ with GAPDH or U6 as an internal control.

### 2.4 MTT assay

Cells were plated into 96‐well plates at a density of 5 × 10^3^ cells/well and cultured for 24–72 h. Thereafter, 20 µl MTT solution (5 mg/L; Sigma‐Aldrich) was added to each well. After incubation for additional 4 h, 150 μl DMSO (Sigma‐Aldrich) was added to dissolve the formazan crystals. The absorbance of each well was read at 570 nm on an ELx808 microplate reader (BioTek Instruments, Inc.).

### 2.5 Colony formation assay

Cells (1,000 cells/well) were suspended and seeded onto six‐well plates. After 14 days, the medium was removed, and the colonies were fixed with methanol and stained with 0.1% crystal violet at room temperature. The number of colonies containing >10 cells was counted manually.

### 2.6 Transwell assay

Cells in serum‐free medium were placed into the uncoated or matrigel‐coated upper chamber of transwell plates (8 μm pore size; Corning Inc.) at a density of 2 × 10^4^ cells/well. Medium containing 10% FBS was added into the lower chamber as a chemoattractant. After 24 h of incubation, the cells in the upper chamber were wiped off, and the cells that passed through the filter were fixed with methanol and stained with 0.1% crystal violet. The stained cells were counted with five random fields.

### 2.7 Western blot analysis

Total protein was extracted using RIPA lysis buffer (Beyotime). Equal amounts of protein samples were separated by SDS‐polyacrylamide gel electrophoresis, and then transferred onto polyvinylidene fluoride (PVDF) membranes (Millipore). After blocking in 5% nonfat milk for 1 h, the membranes were incubated with the specific primary antibodies overnight at 4℃, followed by incubation with the HRP‐conjugated secondary antibody for 1 h at room temperature. The protein bands were visualized using an enhanced chemiluminescent detection kit (Beyotime). GAPDH was used as a protein‐loading control.

### 2.8 Dual‐luciferase reporter assay

The circVANGL1 sequence containing the putative target sites for miR‐150‐5p was amplified by PCR and inserted into the psiCHECK‐2 luciferase reporter vector (Promega). The mutant constructs were generated using the GeneTailor™ Site‐Directed Mutagenesis System (Invitrogen). The luciferase reporters were co‐transfected into cells with miR‐150‐5p mimics or miR‐NC using Lipofectamine 2000. Cells were lysed 48 h after transfection, and the luciferase activities were measured with the Dual‐Luciferase Reporter Assay System (Promega).

### 2.9 RNA immunoprecipitation (RIP) assay

RIP assay was carried out using the EZ‐Magna RIP™ RNA‐Binding Protein Immunoprecipitation Kit (Millipore). Cells were lysed in RNA lysis buffer, and the cell lysates were then incubated with RIP buffer containing magnetic beads conjugated to human anti‐Ago2 antibody (Millipore) or mouse IgG (Millipore). Finally, the RNAs in the magnetic bead‐binding complexes were purified and subjected to RT‐qPCR analysis.

### 2.10 Statistical analysis

All statistical analyses were performed using GraphPad Prism 6.0 software (GraphPad Software Inc.) and SPSS 18.0 software (SPSS Inc.). Continuous data are expressed as mean ± standard deviation (SD) and compared using Student's *t* test or one‐way ANOVA. The differences between groups were undertaken using Student's *t* test or one‐way ANOVA. Statistical significance was set at *p *< 0.05.

## 3 RESULTS

### 3.1 CircVANGL1 is up‐regulated in melanoma

**FIGURE 1:**
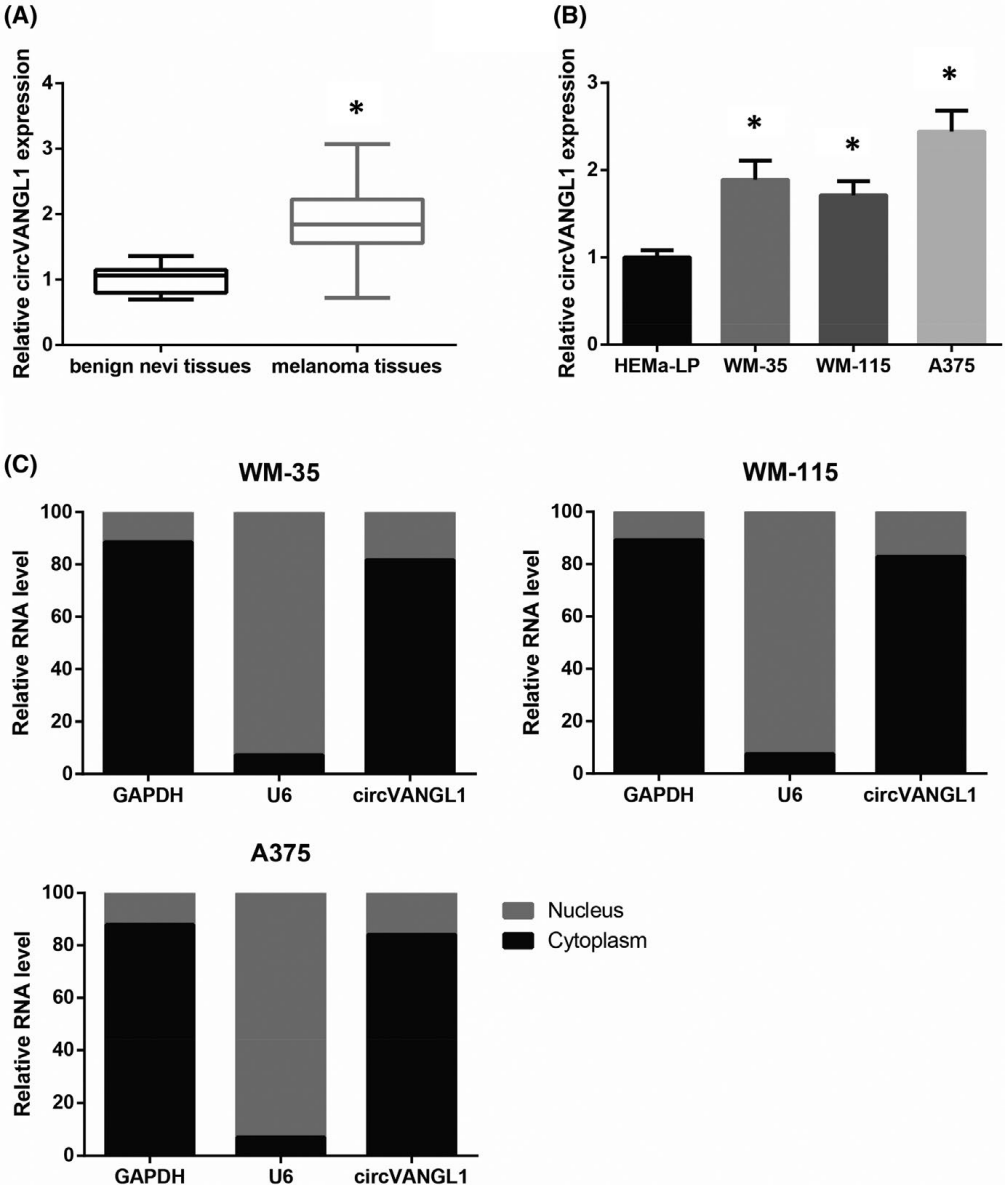
circVANGL1 is up‐regulated in melanoma. A, The expression of circVANGL1 in melanoma tissues and benign nevi tissues, detected by RT‐qPCR analysis. B, The expression of circVANGL1 in melanoma cell lines and HEMa‐LP cells. C, The subcellular localization of circVANGL1 in melanoma cells. *p < 0.05 vs. benign nevi tissues or HEMa‐LP cells

Through RT‐qPCR analysis, we observed that circVANGL1 expression was notably increased in melanoma tissues, compared with benign nevi tissues (Figure 1A). In addition, circVANGL1 was also up‐regulated in melanoma cell lines (WM‐35, WM‐115 and A375), compared with normal HEMa‐LP cells (Figure 1B). Moreover, we observed that circVANGL1 was predominantly localized in the cytoplasm of WM‐35, WM‐115 and A375 cells (Figure 1C).

We then investigated the correlation between circVANGL1 expression and clinicopathological characteristics in 69 melanoma patients. According to the median circVANGL1 expression level, these patients were allocated into two groups, including low expression group (*n* = 39) and high expression group (*n* = 30). As exhibited in Table 1, melanoma patients with high circVANGL1 expression exhibited a close association with larger tumour thickness (*p *= 0.035), advanced TNM stage (*p *= 0.006) and lymphatic metastasis (*p *= 0.031).

**TABLE 1:** Association between clinicopathological characteristics and circVANGL1 expression in 69 melanoma patients

Characteristics	Total number	circVANGL1 expression		*p* Value
		High (*n* = 30)	Low (*n* = 39)	
Age (years)				
<60	39	15	24	0.338
≥60	30	15	15	
Gender				
Male	48	22	26	0.551
Female	21	8	13	
Tumour thickness (mm)				
<1.0	33	10	23	0.035
≥1.0	36	20	16	
Tumour site				
Extremities	18	7	11	0.901
Trunk	40	18	22	
Head and neck	11	5	6	
Histologic type				
Superficial spreading	21	7	14	0.528
Nodular	15	7	8	
Acral lentiginous	33	16	17	
TNM stage				
I–II	29	7	22	0.006
III‐IV	40	23	17	
Lymphatic metastasis				
No	26	7	19	0.031
Yes	43	23	20	

### 3.2 CircVANGL1 promotes melanoma cell proliferation and invasion

**FIGURE 2:**
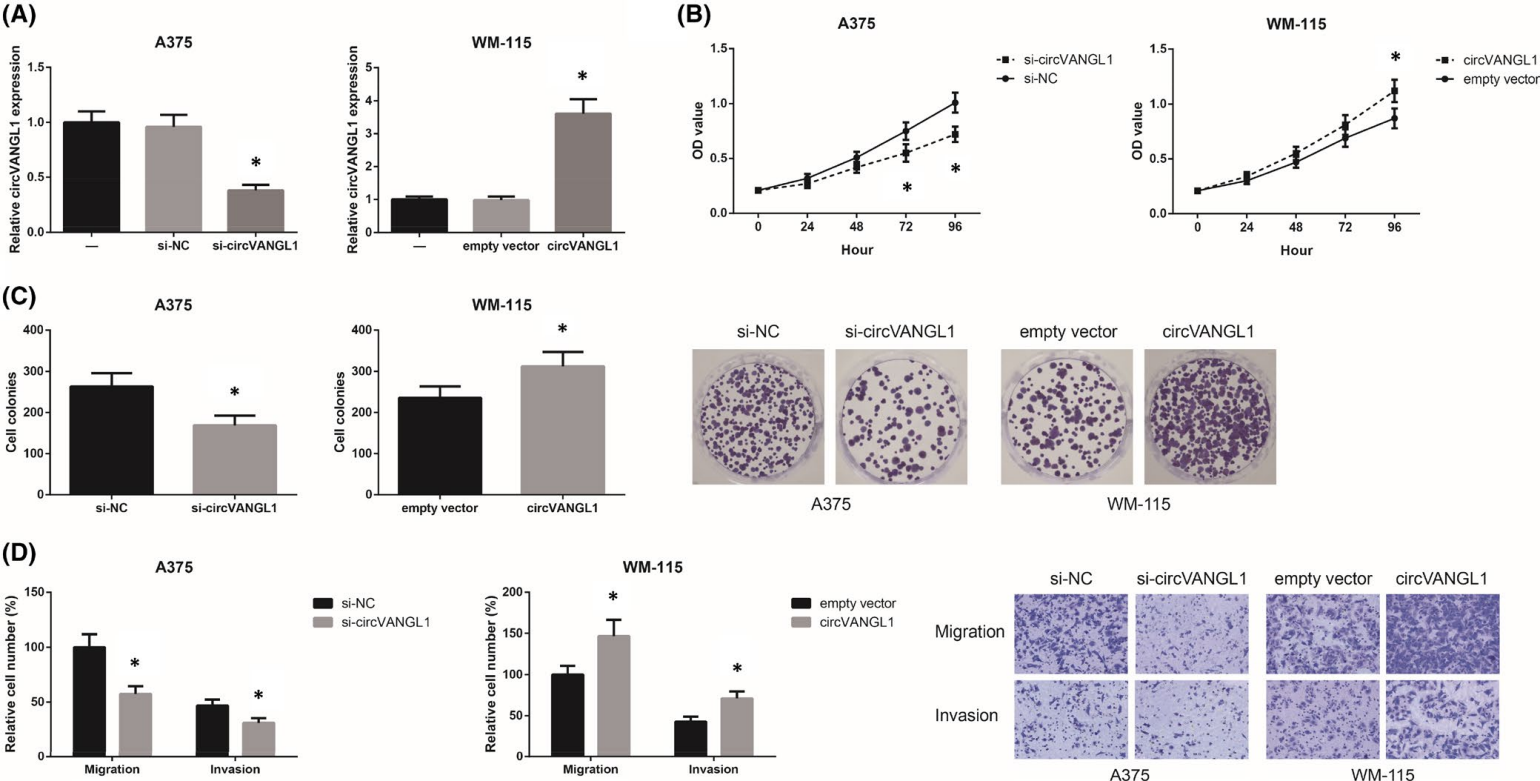
circVANGL1 promotes melanoma cell proliferation and invasion. A, The expression of circVANGL1 in melanoma cells after transfection. B, The proliferation of melanoma cells after transfection, detected by MTT assay. C, The clonogenic ability of melanoma cells after transfection, detected by colony formation assay. D, The migration and invasion of melanoma cells after transfection, detected by transwell assay. *p < 0.05 vs. si‐NC or empty vector‐transfected cells

Functional assays were further carried out to validate the role of circVANGL1 in melanoma progression. As shown in Figure 2A, using siRNA targeting the back‐splicing sequence, we effectively knocked‐down circVANGL1 in A375 cells. We also succeeded in overexpressing circVANGL1 in WM‐115 cells. The results of MTT assay revealed that the proliferation of A375 cells was markedly suppressed by circVANGL1 knockdown, while circVANGL1 overexpression caused a significant promotion in the proliferation of WM‐115 cells (Figure 2B). Besides, as demonstrated in Figure 2C, circVANGL1 knockdown markedly reduced the number of colonies formed by A375 cells, while the clonogenic capacity of WM‐115 cells was enhanced by circVANGL1 overexpression. Moreover, as indicated by transwell assay, circVANGL1 knockdown also notably inhibited the migratory and invasive abilities of A375 cells, whereas these abilities were enhanced by circVANGL1 overexpression in WM‐115 cells (Figure 2D).

### 3.3 CircVANGL1 directly binds to miR‐150‐5p in melanoma

**FIGURE 3:**
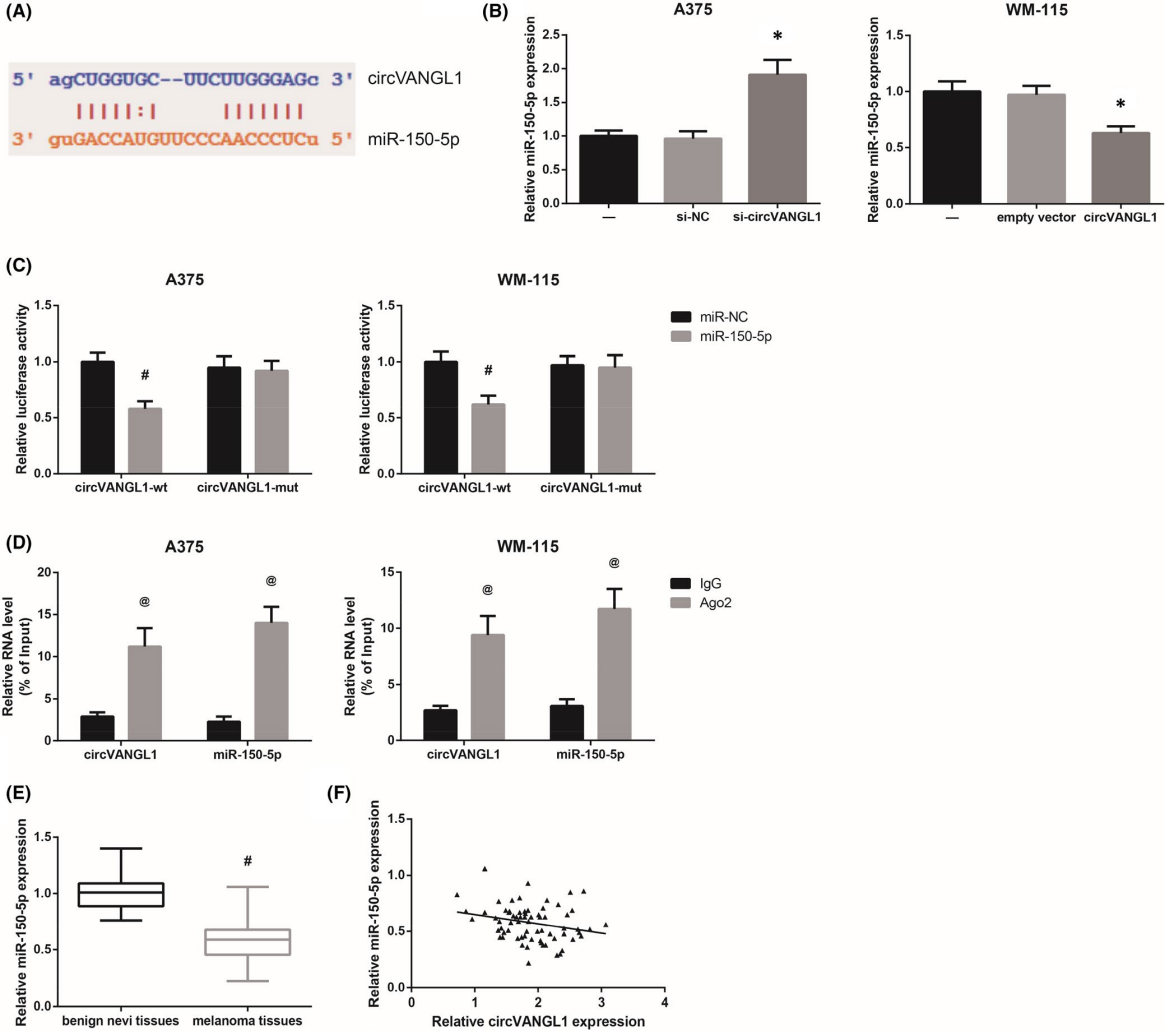
circVANGL1 directly binds to miR‐150‐5p in melanoma. A, The putative binding sites between circVANGL1 and miR‐150‐5p. B, The expression of miR‐150‐5p in melanoma cells after transfection. C, The luciferase activities of recombinant reporters in melanoma cells after transfection. D, The enrichment of circVANGL1 and miR‐150‐5p in melanoma cells, detected by RIP assay. E, The expression of circVANGL1 in melanoma tissues and benign nevi tissues. F, Pearson correlation analysis of circVANGL1 and miR‐150‐5p expression in melanoma tissues. *p < 0.05 vs. si‐NC or empty vector‐transfected cells; ^#^ *p *< 0.05 vs. miR‐NC‐transfected cells or benign nevi tissues; ^@^ *p *< 0.05 vs. IgG immunoprecipitates

Since circVANGL1 was mainly located in the cytoplasm, we therefore speculated that circVANGL1 might serve as microRNA sponges in melanoma. By using the online software program Starbase (http://starbase.sysu.edu.cn/index.php), we noticed that circVANGL1 formed complementary base pairing with miR‐150‐5p (Figure 3A). We also found that circVANGL1 knockdown increased, while circVANGL1 overexpression decreased miR‐150‐5p expression in A375 and WM‐115 cells, respectively (Figure 3B). Dual‐luciferase reporter assay further indicated a significant reduction in the luciferase activities of circVANGL1‐wt after co‐transfection with miR‐150‐5p mimics in A375 andWM‐115 cells, but the luciferase activities of circVANGL1‐mut were not obviously affected (Figure 3C). Ago2, a critical component of RNA‐induced silencing complex (RISC), serves as a key regulator of miRNA functions ^11^. To determine whether circVANGL1 and miR‐150‐5p are in the same RISC, RIP assay was then carried out, and the results demonstrated that the levels of circVANGL1 and miR‐150‐5p were remarkably higher in Ago2 immunoprecipitates in A375 and WM‐115 cells, compared with IgG immunoprecipitates (Figure 3D). Furthermore, miR‐150‐5p expression was significantly decreased in melanoma tissues, compared with benign nevi tissues (Figure 3E), and a negative correlation was observed between the expression of circVANGL1 and miR‐150‐5p in melanoma tissues (Figure 3F).

### 3.4 MiR‐150‐5p restoration blocks the oncogenic role of circVANGL1 in melanoma cells

**FIGURE 4:**
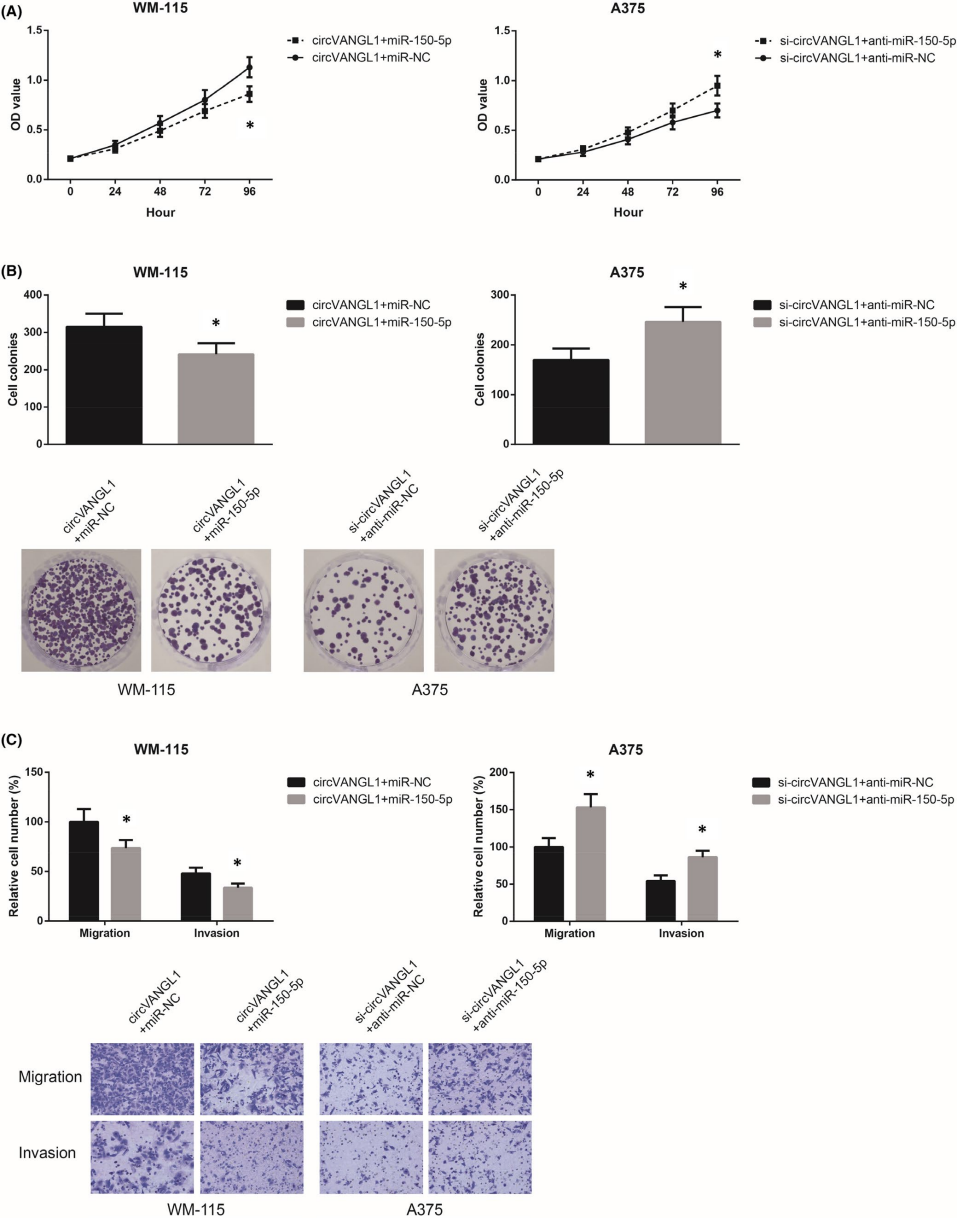
miR‐150‐5p restoration blocks the oncogenic role of circVANGL1 in melanoma cells. A, The proliferation of melanoma cells after transfection, detected by MTT assay. B, The clonogenic ability of melanoma cells after transfection, detected by colony formation assay. C, The migration and invasion of melanoma cells after transfection, detected by transwell assay. *p < 0.05 vs. miR‐NC or anti‐miR‐NC‐transfected cells

We then performed rescue experiments to verify whether miR‐150‐5p was involved in the functions of circVANGL1 in melanoma. As demonstrated in Figure 4A,B, the enhanced proliferation and clonogenic capacity of circVANGL1‐overexpressing WM‐115 cells were notably blocked by co‐transfection with miR‐150‐5p mimics. MiR‐150‐5p restoration also remarkably suppressed the migratory and invasive abilities of circVANGL1‐overexpressing WM‐115 cells (Figure 4C). In addition, we confirmed that the effects of circVANGL1 knockdown in A375 cells were largely diminished by miR‐150‐5p inhibition.

### 3.5 CircVANGL1 knockdown inhibits EMT of TGF‐β‐treated melanoma cells

**FIGURE 5:**
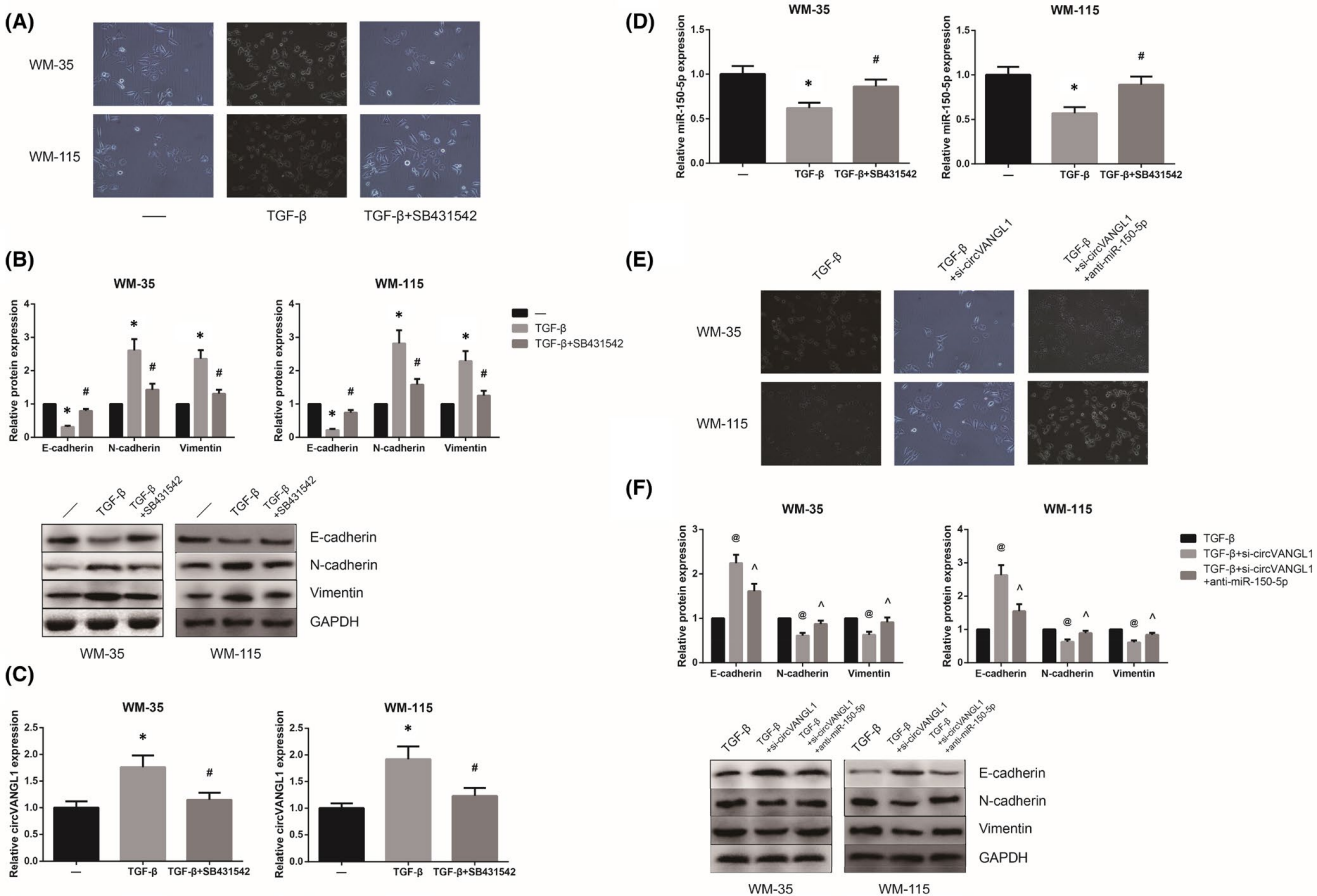
circVANGL1 knockdown inhibits EMT of TGF‐β‐treated melanoma cells. A, The morphology of melanoma cells after treatments. B, The expression of EMT‐related proteins in melanoma cells after treatments, detected by Western blot analysis. C, The expression of circVANGL1 in melanoma cells after treatments. D, The expression of miR‐150‐5p in melanoma cells after treatments. E, The morphology of melanoma cells after treatments. F, The expression of EMT‐related proteins in melanoma cells after treatments. *p < 0.05 vs. cells without TGF‐β treatment; ^#^ *p *< 0.05 vs. TGF‐β‐treated cells without SB431542 treatment; ^@^ *p *< 0.05 vs. cells without transfection; ^*p *< 0.05 vs. si‐circVANGL1‐transfected cells without miR‐150‐5p inhibition

As shown in Figure 5A,B, WM‐35 and WM‐115 cells treated with TGF‐β exhibited a spindle‐shaped morphology, accompanied by the loss of E‐cadherin and up‐regulation of N‐cadherin and Vimentin. We also observed that the increased circVANGL1 expression and decreased miR‐150‐5p expression in TGF‐β‐treated WM‐35 and WM‐115 cells were obviously reversed by SB431542, a TGF‐β receptor antagonist (Figure 5C,D). Moreover, as exhibited in Figure 5E,F, the TGF‐β‐induced EMT‐related characteristics were also markedly diminished by circVANGL1 knockdown, and these effects were obviously blocked by miR‐150‐5p inhibition.

## 4 DISCUSSION

Melanoma is the most dangerous type of skin cancer, and its aetiology is a complex process involving multiple environmental, phenotypic and genetic factors. In recent years, many circRNAs have been identified to function as important drivers of tumorigenesis or tumour suppressors in melanoma.^12^ The oncogenic functions of circVANGL1 were previously reported, and in the present study, we aimed to investigate the functional role of circVANGL1 in melanoma progression.

CircVANGL1 expression levels were observed to be significantly increased in clinical melanoma tissues and cell lines. We then conducted a series of functional experiments and demonstrated that circVANGL1 knockdown remarkably suppressed, while circVANGL1 overexpression enhanced the proliferation, migration and invasion abilities of melanoma cells. EMT is identified as a major determinant of melanoma metastasis, and therefore, modulation of EMT is a potential therapeutic strategy for reducing the aggressive progression of metastatic melanoma.^13^, ^14^ TGF‐β is a secreted cytokine that stimulates tumour cells to undergo EMT.^15^ This study further showed that TGF‐β treatment enhanced EMT in melanoma cells, and the EMT‐related characteristics were obviously diminished by circVANGL1 knockdown.

Next, we explored the mechanisms by which circVANGL1 serves as an oncogene in melanoma. It has been widely recognized that circRNAs can function as microRNA sponges, indicating that circRNAs bind to miRNAs and consequently repress their functions.^16^, ^17^ Bioinformatic tools identified that miR‐150‐5p harbours a complementary sequence in circVANGL1 sequence. Previous studies have reported the inhibitory role of miR‐150‐5p in melanoma progression,^18^, ^19^ and through experimental validations, this study verified that circVANGL1 could directly interact with miR‐150‐5p and negatively regulated its expression in melanoma. By rescue experiments, we further confirmed that miR‐150‐5p restoration could block the oncogenic role of circVANGL1 in melanoma cells.

In conclusion, the findings of our study clearly demonstrated that circVANGL1 is up‐regulated in melanoma and that it can enhance the malignant traits of melanoma cells partly by sponging miR‐150‐5p, indicating that targeting circVANGL1/miR‐150‐5p axis may be a promising therapeutic strategy for melanoma patients in the future.

## CONFLICT OF INTEREST

None.

## AUTHOR CONTRIBUTION

**Jin Wu:** Conceptualization (equal); Funding acquisition (equal); Investigation (equal); Methodology (equal); Supervision (equal); Validation (equal); Writing‐original draft (equal); Writing‐review & editing (equal). **Shaolong Leng:** Conceptualization (equal); Investigation (equal); Methodology (equal); Validation (equal); Writing‐original draft (equal). **Chongchao Hou:** Conceptualization (equal); Investigation (equal); Methodology (equal); Resources (equal); Writing‐original draft (equal). **Laiming Mo:** Conceptualization (equal); Data curation (equal); Investigation (equal); Methodology (equal); Validation (equal); Writing‐original draft (equal). **Xue Xie:** Conceptualization (equal); Formal analysis (equal); Funding acquisition (equal); Investigation (equal); Methodology (equal); Validation (equal); Writing‐original draft (equal). **Ling Wang:** Conceptualization (equal); Formal analysis (equal); Funding acquisition (equal); Investigation (equal); Methodology (equal); Validation (equal); Writing‐original draft (equal). **Hongfeng Zhou:** Formal analysis (equal); Methodology (equal); Writing‐original draft (equal). **Yunsheng Xu:** Investigation (equal); Methodology (equal); Resources (equal); Writing‐review & editing (equal).
